# 315. A Multi-center Study to Describe Obese Pediatric Patients with COVID-19 Across the United States

**DOI:** 10.1093/ofid/ofab466.517

**Published:** 2021-12-04

**Authors:** Roukaya Al Hammoud, James Murphy, Gabriela P Del Bianco, Gloria Heresi, Michael L Chang

**Affiliations:** 1 The University of Texas Health Science Center, McGovern Medical Center, Houston, Texas; 2 The University of Texas Health Science Center at Houston, Houston, TX; 3 UT Health McGovern Medical School, Houston, TX

## Abstract

**Background:**

Obesity is linked to increased risk of complications and is reported to be the most common underlying condition for severely ill SARS-CoV-2 infected individuals. Therefore, we aim further to explore the clinical outcomes of obese children with COVID-19.

**Methods:**

Data were from the Pediatric COVID-19 Case Registry, which includes any patient < 21 years of age diagnosed with COVID-19 at 170 instructions across the United States. A total of 778 COVID-19 positive non-immunocompromised hospitalized patients aged 24 months or older were included. Patients were assigned as obese or non-obese based on BMI as reported from medical records referenced to CDC BMI by gender and age classification (https://www.cdc.gov/growthcharts/clinical_charts.htm).

**Results:**

Patients meeting inclusion criteria included 56% not obese and 44% obese. Compared to matched US population, obese children and adolescents appeared in this database at a rate of 2.3 times their frequency in the population. Obese patients were more likely to be Hispanic and older, symptomatic, have abnormal radiological findings, and require oxygen and ICU admission. Mortality, in this analysis, was similar across the groups.

Demographic and clinical characteristics. NS: Not significant *within seven days of COVID diagnosis ***mild: no need for supplemental oxygen; moderate: need for supplemental oxygen and severe: need for mechanical ventilation.

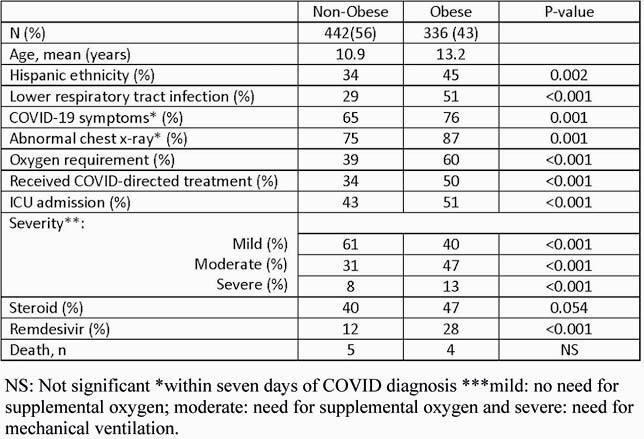

**Conclusion:**

The incidence of obesity in hospitalized COVID children is higher than that of the general population (34% vs. 19%), highlighting obesity as an important risk factor for hospitalization associated with SARS-CoV-2 infected. Therefore, obese children and adolescents with COVID should be prioritized for COVID immunization and managed aggressively, given their significant COVID morbidity.

**Disclosures:**

**All Authors**: No reported disclosures

